# Comparative study of targeted next-generation sequencing and traditional pathogen detection methods in lower respiratory tract infections: impact on patient outcomes

**DOI:** 10.3389/fmicb.2026.1802040

**Published:** 2026-05-08

**Authors:** Zhengyuan An, Xiaoping Guo, Xu Lu, Haoming Tian, Shengfang Zhang, Zhirong Mao, Shirong Fan, Xu Zhang, Pingping Tao, Zijuan Li, Jun Su, Zuyi Chen

**Affiliations:** 1 Department of Medical Laboratory, People's Hospital of Dejang, Tongren, Guizhou, China; 2 Department of Laboratory Medicine, Affiliated Hospital of Zunyi Medical University, Zunyi, China

**Keywords:** comparative effectiveness, pathogen detection, patient outcomes, respiratory tract infections, targeted next-generation sequencing

## Abstract

**Background:**

Lower respiratory tract infections (LRTIs) are major health problem worldwide. Accurate pathogen identification using conventional microbiological tests (CMTs) is challenging. Targeted next-generation sequencing (tNGS) is a promising diagnostic approach; however, its performance compared with that of CMTs, and its impact on patient outcomes require further investigation.

**Methods:**

This retrospective study enrolled 562 patients with suspected lower respiratory infections from 29 departments during 2023–2025. Bronchoalveolar lavage fluid samples from all patients underwent parallel testing with both tNGS and CMTs. The primary outcomes were pathogen detection rates (tNGS vs. CMTs), turnaround time, and clinical outcomes. Secondary outcomes comprised antibiotic modification rates, length of hospital stay, 28-day mortality, and intensive care unit admission rates. Multivariable logistic regression and propensity score adjustment were used to assess the independent impact of tNGS-guided therapy on patient outcomes.

**Results:**

The pathogen detection rate was significantly higher with tNGS than with CMTs (89.5% vs. 62.1%, *p* < 0.001; McNemar test). tNGS was more sensitive than CMTs for detecting special pathogens (36.8% vs. 8.9%, *p* < 0.001), viruses (83.5% vs. 18.3%, *p* < 0.001), and fungi (73.5% vs. 31.7%, *p* < 0.001). Among 314 patients with tNGS-adopted management, 68.8% received antibiotic modifications. In the adjusted analysis, tNGS-guided therapy was associated with a reduced length of hospital stay (adjusted *β* = −2.3 days, *p* = 0.003) and lower 28-day mortality (adjusted odds ratio: 0.42, *p* = 0.032).

**Conclusion:**

tNGS is more sensitive than CMTs at detecting all categories of pathogens and when results are used to guide clinical management, tNGS is associated with improved clinical outcomes including reduced mortality and shorter hospital stays.

## 1 Introduction

Lower respiratory tract infections (LRTIs) continue to pose a substantial threat to global health, ranking as the fourth leading cause of death worldwide and accounting for approximately 2.5 million deaths annually (Li et al., 2025; Gao et al., 2024). Despite significant advances in medical care, the timely and accurate identification of causative pathogens remains a fundamental challenge in clinical practice. Traditional microbiological diagnostic methods, including culture-based techniques, microscopy, serology, and targeted polymerase chain reaction (PCR), often do not provide comprehensive or rapid pathogen identification (Liang et al., 2022; Zheng et al., 2021). These limitations frequently result in empirical broad-spectrum antibiotic use, which contributes to antimicrobial resistance and may lead to suboptimal patient outcomes (Diao et al., 2022).

The complexity of lower respiratory infections is further compounded by the diverse array of potential pathogens, including bacteria, fungi, viruses, and atypical organisms such as mycobacteria. Mixed infections involving multiple pathogen types are increasingly recognized, particularly in immunocompromised patients and those with severe pneumonia (Liu et al., 2023; Lv et al., 2024). Conventional microbiological tests (CMTs) often lack the sensitivity and breadth to detect these diverse pathogens, especially fastidious organisms, rare pathogens, and those affected by prior antibiotic therapy (Elbehiry et al., 2025). Moreover, the prolonged turnaround time (TAT) associated with culture-based methods, which often require 48–72 h or longer, delays targeted antimicrobial therapy and may have an adverse effect on patient outcomes (Chen et al., 2020).

Metagenomic next-generation sequencing (mNGS) has emerged as a revolutionary diagnostic tool that enables unbiased, culture-independent direct detection of a wide spectrum of pathogens from clinical specimens (Yin et al., 2025; Gaston et al., 2022). mNGS is more sensitive than traditional approaches, particularly for detecting viruses, fungi, and atypical pathogens (Deng et al., 2022; Zhang et al., 2022). However, clinical implementation of mNGS faces challenges including high cost, interference from host nucleic acids, and difficulties in distinguishing pathogens from commensals (Hu et al., 2025; Long et al., 2024).

To address these limitations, targeted next-generation sequencing (tNGS) has been developed as an alternative approach that combines targeted amplification with high-throughput sequencing technology. Its targeted amplification selectively enriches pathogen nucleic acids, minimizing host nucleic acid interference without additional host DNA depletion steps, and features broad pathogen coverage, high detection sensitivity, and a significantly shorter turnaround time compared with mNGS (Yin et al., 2024). Recent studies have demonstrated that tNGS has comparable diagnostic performance to that of mNGS while offering practical advantages for clinical implementation (Xu et al., 2025; Sun et al., 2024). However, few head-to-head comparisons between tNGS and CMTs in large patient cohorts are available, and the impact of tNGS-guided management on patient outcomes such as mortality, length of stay, and antibiotic stewardship has not been adequately investigated.

Previous studies have focused primarily on diagnostic performance metrics such as sensitivity and specificity but have not comprehensively evaluated the clinical utility of tNGS in terms of treatment modifications and patient outcomes. Understanding whether superior pathogen detection translates into improved clinical outcomes is essential for justifying the higher costs and resources required for tNGS implementation. Furthermore, methodologically rigorous comparative studies with adjustment for confounding variables are needed to establish causal relationships between tNGS use and clinical outcomes.

This study aimed to address these knowledge gaps by: (1) comparing pathogen detection rates between tNGS and CMTs using paired samples from the same patients; (2) evaluating the impact of tNGS on clinical decision-making and antibiotic management; (3) assessing the relationship between tNGS-guided therapy and patient outcomes, including mortality, intensive care unit (ICU) admission, and length of stay; and (4) identifying factors associated with successful clinical outcomes.

## 2 Materials and methods

### 2.1 Study design and patients

This retrospective, single-center observational cohort study was conducted at a tertiary care hospital from January 1, 2023, to December 30, 2025. The study protocol was reviewed and approved by the institutional ethics committee (Ethics approval number: 2025048). The requirement for informed consent was waived owing to the retrospective study design. All patient medical data were de-identified and analyzed using unique medical record numbers, ensuring that no personally identifiable information was included in the statistical analysis or reporting.

The inclusion criteria for this study were: (1) patients aged ≥1 year with suspected LRTI based on clinical presentation, radiological findings, and laboratory abnormalities; (2) availability of bronchoalveolar lavage fluid (BALF) samples collected during the hospital stay; (3) completion of both tNGS testing and at least one CMT on the same BALF specimen collected simultaneously or a paired sample collected within 24 h; and (4) availability of complete clinical data including demographics, laboratory results, treatment records, and outcome information including vital status at 28 days. The exclusion criteria included: (1) patients with incomplete medical records or missing key clinical data; (2) patients with samples of inadequate quality (ineligibility criteria: BALF recovery rate < 30%, red blood cell proportion > 10%, squamous epithelial cell proportion > 1%) or quantity for parallel testing; and (3) patients transferred to other facilities before outcome assessment.

Baseline severity was assessed using CURB-65 scores for adults and modified criteria for pediatric patients. Comorbidities including chronic respiratory disease, diabetes mellitus, cardiovascular disease, immunosuppression, and malignancy, were systematically recorded.

Patients were classified into two groups based on their clinical management: (1) tNGS-Adopted group: patients in whom tNGS results were used to guide antimicrobial therapy, defined as documented changes in antimicrobial management explicitly attributed to tNGS findings in the medical records, or physician documentation confirming that tNGS results informed treatment decisions; and (2) a Non-tNGS-Adopted group: patients whose antimicrobial management was based solely on CMT results or empirical therapy without documented consideration of tNGS findings. The classification was performed through systematic chart review by two independent investigators blinded to clinical outcomes, with disagreements resolved by a third senior physician. This classification was made retrospectively based on medical record documentation and was independent of the microbiological test results. Importantly, all 562 patients underwent both tNGS and CMTs testing on paired BALF samples regardless of group assignment; the group classification reflected whether clinicians subsequently used tNGS results to guide treatment decisions in clinical practice.

### 2.2 Sample collection and processing

Bronchoalveolar lavage was performed by experienced pulmonologists following standardized protocols. Prior to the procedure, patients underwent comprehensive evaluation to exclude contraindications, and written informed consent was obtained. A flexible bronchoscope was advanced through the nasopharynx or oropharynx into the target bronchial segment identified by chest imaging findings, under local anesthesia and conscious sedation when appropriate. The bronchoscope was wedged into the subsegmental bronchus, and sterile normal saline (37 °C) was instilled in aliquots of 20–40 mL, with 2–3 lavages performed per site. The aspirated fluid was immediately collected into sterile containers.

On receipt in the laboratory, the BALF samples were divided into multiple aliquots for parallel testing: (1) one aliquot was immediately sent for conventional microbiological testing, including bacterial culture, fungal culture, acid-fast bacilli smear and culture, and viral detection as clinically indicated; (2) a second aliquot of 5 mL was reserved for tNGS testing and processed within 24 h; (3) additional aliquots were used for cell count, differential, and biochemical analysis. Critically, sample splitting and processing were performed simultaneously to ensure valid comparison between methods.

### 2.3 tNGS methodology

The tNGS assay used in this study tested for a comprehensive panel designed to detect approximately 225 common respiratory pathogens, including bacteria, fungi, viruses, mycobacteria, and other atypical microorganisms (see Supplementary material 1 for details). The methodology combined ultra-multiplex PCR with high-throughput sequencing to enable simultaneous detection of both DNA and RNA pathogens. Nucleic acid extraction was performed using the Pathogenic RNA/DNA Kit (Guangzhou Magen Biotechnology Co., Ltd., Guangzhou, China) optimized for clinical respiratory specimens. Following extraction, samples underwent reverse transcription for RNA viruses and then multiplex PCR amplification targeting specific conserved and variable regions of pathogen genomes. Library preparation was performed using the Respiratory Pathogenic Nucleic Acids Multiple Detection kit (Guangzhou Jinqirui Biotechnology Co., Ltd., Guangzhou, China), which included enzymatic fragmentation, end-repair, adapter ligation, and indexing. Sequencing was performed using the KM MiniSeqDx-CN platform (Illumina, Inc., San Diego, CA, USA) with paired-end reads, generating approximately 5–10 million reads per sample. Bioinformatic analysis involved quality filtering of raw reads, alignment to comprehensive pathogen databases, and taxonomic classification. The entire workflow from sample receipt to final report typically took 20–24 h. A positive tNGS result was defined as detection of one or more pathogens with read counts above the validated threshold (typically ≥50 unique reads for bacteria/fungi, ≥10 reads for viruses). The limit of detection for representative bacteria is provided in Supplementary material 2 for details.

### 2.4 CMT methodology

CMTs were performed according to standard clinical laboratory procedures and included multiple complementary approaches. Bacterial culture was conducted using blood agar, chocolate agar, and MacConkey agar plates (Autobio Diagnostics Co., Ltd., Zhengzhou, China), with incubation at 35 °C in ambient air and 5% CO_2_ for up to 5 days. Bacterial identification was performed using matrix-assisted laser desorption ionization time-of-flight mass spectrometry (MALDI-TOF MS) (Zybio. Inc., Chongqing, China), covering 3,585 bacteria and 1,432 fungi. Fungal culture was performed using Sabouraud dextrose agar with incubation at 30 °C for up to 4 weeks. Acid-fast bacilli smears were performed using Ziehl–Neelsen staining method, and mycobacterial cultures were inoculated on Löwenstein–Jensen medium with incubation at 37 °C for up to 8 weeks. Detection of *Mycobacterium tuberculosis* (*M. tuberculosis*) complex also included GeneXpert MTB/RIF assay (Xiamen Zishan Biotechnology Co., Ltd.) when clinically indicated. Viral testing included multiplex respiratory virus PCR panels (Suzhou Tianlong Biotechnology Co., Ltd., Suzhou, Jiangsu, China) targeting common respiratory viruses (such as influenza virus A and B, adenovirus, and respiratory syncytial virus). *Mycoplasma pneumoniae* testing included multiplex respiratory virus PCR panels (Suzhou Tianlong Biotechnology Co., Ltd.). Galactomannan antigen testing and (1,3)-β-D-glucan assays (Zhanjiang A&C Biological Ltd., Zhanjiang, Guangdong, China) were conducted for suspected invasive fungal infections. A positive CMT result was defined as growth of organisms in culture, or positive molecular/antigen tests.

### 2.5 Data collection and outcome definitions

Comprehensive clinical and laboratory data were extracted from electronic medical records using a standardized data collection form. All 562 patients underwent both tNGS and CMTs testing on paired BALF samples to enable comparative diagnostic evaluation; subsequent patient classification into tNGS-Adopted versus Non-tNGS-Adopted groups was based on the clinical utilization of the results as described in Section 2.1. The following data elements were systematically collected: (1) demographic information including age, sex, admission date, and discharge date; (2) clinical severity assessment including CURB-65 scores, need for mechanical ventilation, and vasopressor support; (3) comorbidities and immunosuppression status; (4) laboratory parameters including complete blood count, inflammatory markers (C-reactive protein [CRP], procalcitonin [PCT]), and organ function tests; (5) radiological findings from chest imaging; (6) microbiological test results from both tNGS and CMTs with exact dates and times of reporting; (7) antimicrobial therapy details including empirical regimen, modifications based on test results, duration, and de-escalation; and (8) clinical outcomes.

Primary outcome measures were: (1) overall pathogen detection rates (any pathogen detected) compared between tNGS and CMTs using paired analysis; (2) category-specific detection rates for bacteria, fungi, viruses, and special pathogens; (3) detection of mixed infections (≥2 pathogen categories); and (4) TAT from sample collection to result reporting.

Secondary outcome measures included (Liang et al., 2022; Lv et al., 2024; Elbehiry et al., 2025): (1) clinical adoption of tNGS results, with patient classification into tNGS-Adopted versus Non-tNGS-Adopted groups performed as described in Section 2.1 based on systematic review of medical documentation; (2) antibiotic modification rates including escalation (addition of antimicrobials or switch to broader spectrum), de-escalation (narrowing of spectrum or stopping antimicrobials), or change to targeted therapy; (3) length of hospital stay from admission to discharge; (4) 28-day all-cause mortality; (5) ICU admission or transfer during hospitalization; (6) days of antibiotic therapy (DOT) per 100 patient-days; (7) hospital readmission within 30 days; and (8) favorable composite outcome, defined as meeting all three of the following criteria: survival at 28 days AND no ICU admission or transfer during hospitalization AND hospital length of stay ≤14 days. Patients who died within 28 days, required ICU admission/transfer, or had hospital stay >14 days were classified as not achieving favorable composite outcome.

### 2.6 Statistical analysis

Statistical analyses were performed using SPSS software version 25.0 and R version 4.2.0. Categorical variables were presented as frequencies and percentages, whereas continuous variables were expressed as mean ± standard deviation for normally distributed data or median and interquartile range (IQR) for non-normally distributed data.

For the primary comparative analysis of detection rates between tNGS and CMTs, McNemar’s test was used for paired binary outcomes (detected vs. not detected). For each pathogen category, relative risk (RR), risk difference (RD), and 95% confidence intervals (CI) were calculated. The TATs of tNGS and CMTs were compared using the paired Wilcoxon signed-rank test given non-normal distribution. For outcome analyses, patients were stratified into groups based on whether tNGS results were clinically adopted to guide therapy. Baseline characteristics between groups were compared using chi-square tests for categorical variables and t-tests or Mann–Whitney U-tests for continuous variables. To address potential confounding by indication (sicker patients more likely to receive tNGS-guided therapy), we used two complementary analytical approaches: First, multivariable regression models were constructed adjusting for predefined confounders including: (1) age; (2) sex; (3) CURB-65 severity score; (4) immunosuppression status; (5) presence of comorbidities; (6) baseline CRP and PCT levels; (7) days from admission to diagnostic testing; and (8) prior antibiotic exposure. Logistic regression was used for binary outcomes (mortality, ICU admission), and negative binomial regression was used for count outcomes (length of stay, antibiotic days).

Second, propensity score analysis was performed to create balanced comparison groups. Propensity scores for receiving tNGS-adopted therapy were estimated using logistic regression including all baseline variables. Inverse probability-of-treatment weighting was then applied to balance covariates between groups, with standardized mean differences <0.1 considered an adequate balance. Weighted regression models were used to estimate adjusted treatment effects. Multiple imputation was used for missing covariate data (<5% for most variables). All statistical tests were two-tailed, and *p* < 0.05 was considered statistically significant. Sensitivity analyses were performed excluding pediatric patients and restricting the analysis to patients with complete data.

## 3 Results

### 3.1 Patient characteristics

The general information and laboratory results of the patients are detailed in Supplementary material 3. A total of 562 patients meeting the inclusion criteria were included in the study. The cohort comprised 307 male (54.6%) and 255 female (45.4%) patients, with a mean age of 53.9 ± 20.0 years (range: 1–89 years). Patients were managed in a total of 29 different departments, with the majority admitted to the Department of Respiratory and Critical Care Medicine (56.8%), Department of Infectious Diseases (17.4%), or the Department of Respiratory Medicine (11.4%). The study cohort comprised 562 patients with suspected respiratory infections who underwent comprehensive diagnostic evaluation including both tNGS and conventional microbiological testing. Table 1 summarizes the baseline demographic and clinical characteristics of the study population. The mean age was 53.5 ± 19.1 years, with a median of 58 years. Elderly patients (60–80 years) represented the largest demographic group at 41.28% (*n* = 232). Male patients constituted 54.6% (*n* = 307) of the cohort.

**Table 1:** Baseline demographic and clinical characteristics.

Characteristic	Total (*n* = 562)	tNGS-adopted (*n* = 314)	tNGS not-adopted (*n* = 248)	*p*-value
Age (years), mean ± SD	53.5 ± 19.1	56.3 ± 18.0	50.0 ± 20.0	0.001
Male sex, *n* (%)	307 (54.6)	178 (56.7)	129 (52.0)	0.285
CURB-65 score ≥3, *n* (%)	189 (33.6)	128 (40.8)	61 (24.6)	<0.001
Comorbidities, *n* (%)				
Chronic respiratory disease	165 (29.4)	98 (31.2)	67 (27.0)	0.283
Diabetes mellitus	112 (19.9)	71 (22.6)	41 (16.5)	0.076
Immunosuppression	143 (25.4)	94 (29.9)	49 (19.8)	0.006
Malignancy	87 (15.5)	56 (17.8)	31 (12.5)	0.082
Laboratory values				
CRP (mg/L), median (IQR)	68.5 (32–125)	82.0 (45–142)	54.0 (28–98)	<0.001
PCT (ng/mL), median (IQR)	0.52 (0.18–2.31)	0.78 (0.25–3.15)	0.38 (0.12–1.52)	<0.001
Prior antibiotics, *n* (%)	487 (86.7)	279 (88.9)	208 (83.9)	0.091
ICU admission, *n* (%)	156 (27.8)	112 (35.7)	44 (17.7)	<0.001

The tNGS-Adopted group had significantly higher disease severity as evidenced by higher CURB-65 scores (*p* < 0.001), elevated inflammatory markers (CRP and PCT, both *p* < 0.001), and higher rates of ICU admission (*p* < 0.001), indicating more severe illness in patients whose management was guided by tNGS results.

### 3.2 Comparative pathogen detection: tNGS vs. CMTs

Tables 2, 3 present the head-to-head comparison of pathogen detection rates between tNGS and CMTs using paired analysis of the same BALF samples. The overall pathogen detection rates were significantly higher with tNGS than with CMTs (89.5% vs. 62.1%, *p* < 0.001; McNemar test). The RR of detecting any pathogen using tNGS compared with CMTs was 1.44 (95% CI: 1.35–1.54), with an RD of 27.4% (95% CI: 23.1–31.7%).

**Table 2:** Paired comparison of pathogen detection rates: tNGS vs. CMTs.

Pathogen category	tNGS positive *n* (%)	CMT positive *n* (%)	RR (95% CI)	RD (95% CI)	*p*-value^*^
Overall detection	503 (89.5)	349 (62.1)	1.44 (1.35–1.54)	27.4% (23.1–31.7%)	<0.001
Bacteria	497 (88.4)	388 (69.0)	1.28 (1.22–1.35)	19.4% (14.9–23.9%)	<0.001
Fungi	413 (73.5)	178 (31.7)	2.32 (2.05–2.62)	41.8% (37.0–46.6%)	<0.001
Viruses	469 (83.5)	103 (18.3)	4.56 (3.82–5.43)	65.2% (60.9–69.4%)	<0.001
Special pathogens^†^	207 (36.8)	50 (8.9)	4.24 (3.20–5.62)	28.8% (24.5–33.2%)	<0.001
Mixed infections (≥2 categories)	375 (66.7)	98 (17.4)	3.84 (3.20–4.61)	49.5% (44.9–54.0%)	<0.001

^*^McNemar test for paired binary outcome. ^†^Special pathogens include *M. tuberculosis* complex, *Mycoplasma pneumoniae*, and *Chlamydia pneumoniae*. CI, confidence interval; RD, risk difference; RR, relative risk.

**Table 3:** Comparison of the turnaround time of tNGS and CMTs.

Test method	Median time (h)	IQR (h)	Mean time (h)	Range (h)	*p*-value^*^
tNGS	24	20–28	25.3 ± 6.8	18–48	—
Bacterial culture	72	48–96	78.5 ± 24.2	24–120	<0.001
Fungal culture	168	120–336	210.4 ± 98.6	48–672	<0.001
Mycobacterial culture	504	336–840	612.8 ± 245.3	168–1,344	<0.001
Viral PCR panel^†^	48	24–72	52.6 ± 18.9	12–96	<0.001

^*^Paired Wilcoxon signed-rank test compared with tNGS. ^†^The longer turnaround time for viral PCR compared with tNGS primarily reflects batching practices and testing schedules at primary care facilities rather than the actual assay time. IQR, interquartile range; PCR, polymerase chain reaction; tNGS, targeted next-generation sequencing.

The detection advantages of tNGS were most pronounced for viruses (RR: 4.56, 95% CI: 3.82–5.43), special pathogens including *M. tuberculosis* and atypical organisms (RR: 4.24, 95% CI: 3.20–5.62), and fungi (RR: 2.32, 95% CI: 2.05–2.62). For bacterial detection, tNGS also showed significant advantage (RR: 1.28, 95% CI: 1.22–1.35, *p* < 0.001), although the difference was less marked than for the other pathogen categories. Notably, the detection of mixed infections was significantly higher with tNGS than with CMTs (66.7% vs. 17.4%; *p* < 0.001).

Among the 562 paired samples, concordance analysis revealed that: (1) both methods were positive in 315 patients (56.0%); (2) tNGS was positive and CMTs were negative in 188 patients (33.5%); (3) CMTs were positive and tNGS was negative in 34 patients (6.0%); and (4) both tNGS and CMTs were negative in 25 patients (4.4%). The overall agreement rate was 60.5% with a kappa (*κ*) coefficient of 0.18 (95% CI: 0.11–0.25), indicating poor agreement primarily due to tNGS detecting many additional pathogens not detected by CMTs.

The median TAT was significantly shorter for tNGS than for all CMTs combined (Table 3), with results available within 24 h for tNGS compared with 72 h for bacterial culture, 168 h for fungal culture, 48 h for a viral PCR panel, and >500 h for mycobacterial culture (all *p* < 0.001).

### 3.3 Pathogen spectrum detected by tNGS

Among the 503 patients with positive tNGS results, special pathogens (including *M. tuberculosis* complex, *Mycoplasma pneumoniae*, and *Chlamydia pneumoniae*) were detected in 207 patients (36.8% of the total cohort). *M. tuberculosis* complex was the most frequently identified special pathogen, detected in 19.0% of all patients. *Mycoplasma pneumoniae* was the second most common special pathogen. Bacterial pathogens were detected in 497 patients with *Fusobacterium nucleatum* and *Haemophilus influenzae* being most common bacterial pathogens. Fungal pathogens were identified in 413 patients, with *Pneumocystis jirovecii* and *Candida* species being the most common fungal pathogens (collectively ~10%). Viral pathogens were detected in 469 patients, with Epstein–Barr virus being most common viral pathogen, followed by rhinoviruses and SARS-CoV-2. The most frequently identified pathogens detected by tNGS and CMTs are shown in Table 4.

**Table 4:** Top pathogens detected by tNGS and CMTs.

Pathogen	tNGS+ total *n* (%)	CMT+ total *n* (%)	Both+ *n* (%)	tNGS+/CMT − *n* (%)	tNGS−/CMT+ *n* (%)
Special pathogens					
*M. tuberculosis* complex	107 (19.0)	32 (5.7)	28 (5.0)	79 (14.1)	4 (0.7)
*Mycoplasma pneumoniae*	34 (6.0)	12 (2.1)	9 (1.6)	25 (4.4)	3 (0.5)
Bacteria					
*Haemophilus influenzae*	73 (13.0)	48 (8.5)	42 (7.5)	31 (5.5)	6 (1.1)
*Streptococcus pneumoniae*	57 (10.1)	52 (9.3)	45 (8.0)	12 (2.1)	7 (1.2)
*Pseudomonas aeruginosa*	15 (2.7)	12 (2.1)	10 (1.8)	5 (0.9)	2 (0.4)
Fungi					
*Pneumocystis jirovecii*	28 (5.0)	8 (1.4)	6 (1.1)	22 (3.9)	2 (0.4)
*Candida albicans*	35 (6.2)	22 (3.9)	18 (3.2)	17 (3.0)	4 (0.7)
*Aspergillus fumigatus*	14 (2.5)	6 (1.1)	4 (0.7)	10 (1.8)	2 (0.4)
Viruses					
Epstein–Barr virus	86 (15.3)	18 (3.2)	16 (2.8)	70 (12.5)	2 (0.4)
SARS-CoV-2	22 (3.9)	21 (3.7)	20 (3.6)	2 (0.4)	1 (0.2)
Rhinovirus A	24 (4.3)	8 (1.4)	7 (1.2)	17 (3.0)	1 (0.2)

CMT, conventional microbiological test; tNGS, targeted next-generation sequencing.

### 3.4 Clinical impact: antibiotic management

Among the 314 patients in whom tNGS results were clinically adopted, antibiotic modifications were documented in 216 patients. Table 5 summarizes the patterns of antibiotic modifications, comparing patients whose management was and was not based on the tNGS results.

**Table 5:** Antibiotic management modifications.

Antibiotic modification	tNGS-adopted (*n* = 314)	Non-tNGS-adopted (*n* = 248)	*p*-value
Any modification, *n* (%)	216 (68.8)	98 (39.5)	<0.001
Type of modification			
Escalation (broader spectrum)	87 (27.7)	52 (21.0)	0.062
De-escalation (narrower spectrum)	64 (20.4)	28 (11.3)	0.004
Targeted change (same spectrum)	65 (20.7)	18 (7.3)	<0.001
Specific changes			
Anti-tuberculosis therapy initiated	82 (26.1)	8 (3.2)	<0.001
Antifungal therapy added	58 (18.5)	22 (8.9)	0.001
Atypical pathogen coverage added	42 (13.4)	6 (2.4)	<0.001
Antibiotic discontinued	31 (9.9)	12 (4.8)	0.027
Days of therapy (DOT)			
Mean total DOT ± SD	12.8 ± 5.4	14.6 ± 6.2	0.001
Broad-spectrum DOT	6.2 ± 4.1	8.9 ± 5.3	<0.001

DOT, days of therapy; SD, standard deviation; tNGS, targeted next-generation sequencing.

Patients with tNGS-adopted management had significantly higher rates of any antibiotic modification (68.8% vs. 39.5%, *p* < 0.001). Notably, de-escalation occurred more frequently in the tNGS-Adopted group than in the Non-tNGS-Adopted group (20.4% vs. 11.3%, *p* = 0.004). The detection of *M. tuberculosis* by tNGS led to initiation of anti-tuberculosis therapy in 82 patients (26.1%) compared with only 8 patients (3.2%) in the Non-tNGS-Adopted group (*p* < 0.001). Total DOT and broad-spectrum antibiotic exposure were both significantly lower in the tNGS-Adopted group than in the Non-tNGS-Adopted group (*p* < 0.001), despite the tNGS-Adopted group having more severe illness at baseline.

### 3.5 Clinical outcomes: unadjusted analysis

Table 6 presents the unadjusted clinical outcomes comparing patients with tNGS-adopted management versus those without tNGS-guided therapy. Despite the tNGS-Adopted group having significantly more severe illness at baseline (higher CURB-65 scores, ICU admission rates, and higher levels of inflammatory markers), they paradoxically showed better outcomes in several domains. The unadjusted mortality rate was lower (5.7% vs. 10.5%, *p* = 0.036), and length of stay was shorter (8.8 vs. 10.6 days, *p* = 0.001) in the tNGS-Adopted group than in the Non-tNGS-Adopted group. However, the ICU admission rate was higher in the tNGS-Adopted group than in the Non-tNGS-Adopted group (35.7% vs. 17.7%, *p* < 0.001).

**Table 6:** Clinical outcomes: unadjusted comparison.

Outcome	tNGS-adopted (*n* = 314)	Non-tNGS-adopted (*n* = 248)	Unadjusted mean difference (95% CI)	*p*-value
Length of stay (days)				
Mean ± SD	8.8 ± 5.9	10.6 ± 7.1	−1.8 (−2.9 to −0.7)	0.001
Median (IQR)	8 (5–11)	10 (6–13)	−2.0 (−3.0 to −1.0)	0.002^*^
28-day mortality, *n* (%)	18 (5.7)	26 (10.5)	RR: 0.55 (0.31–0.97)	0.036
ICU admission, *n* (%)	112 (35.7)	44 (17.7)	RR: 2.01 (1.49–2.71)	<0.001
ICU length of stay (days)^†^	6.2 ± 4.8	7.9 ± 5.6	−1.7 (−3.2 to −0.2)	0.028
Mechanical ventilation, *n* (%)	78 (24.8)	32 (12.9)	RR: 1.92 (1.33–2.79)	<0.001
Ventilator days^†^	5.8 ± 4.2	7.3 ± 5.1	−1.5 (−2.9 to −0.1)	0.038
30-day readmission, *n* (%)	23 (7.3)	28 (11.3)	RR: 0.65 (0.39–1.09)	0.092
Clinical improvement at day 7^‡^, *n* (%)	246 (78.3)	165 (66.5)	RR: 1.18 (1.07–1.30)	0.001

^*^Mann–Whitney U test; ^†^Among patients requiring ICU/mechanical ventilation; ^‡^Defined as resolution of fever, ≥50% reduction in inflammatory markers, and radiological improvement. CI, confidence interval; IQR, interquartile range; RR, relative risk; SD, standard deviation; tNGS, targeted next-generation sequencing.

### 3.6 Adjusted analysis: impact of tNGS-guided therapy on outcomes

To address confounding by indication and the baseline differences in severity between the tNGS-Adopted and Non-tNGS-Adopted groups, we performed multivariable logistic regression analysis to adjust for predefined potential confounders. Table 7 presents the adjusted associations between tNGS-adopted management and clinical outcomes.

**Table 7:** Multivariable analysis: adjusted impact of tNGS-guided therapy on clinical outcomes.

Outcome	Model 1: multivariable adjusted^†^	*p*-value	Model 2: propensity score weighted^‡^	*p*-value
Length of hospital stay				
Adjusted *β* (95% CI)	−2.3 days (−3.8 to −0.8)	0.003	−2.1 days (−3.6 to −0.6)	0.007
28-day mortality				
Adjusted OR (95% CI)	0.42 (0.19–0.93)	0.032	0.38 (0.16–0.89)	0.026
ICU length of stay (days)^§^				
Adjusted *β* (95% CI)	−1.8 (−3.2 to −0.4)	0.013	−1.6 (−3.1 to −0.1)	0.036
Mechanical ventilation duration (days)^§^				
Adjusted *β* (95% CI)	−1.9 (−3.4 to −0.4)	0.011	−1.7 (−3.3 to −0.1)	0.038
Broad-spectrum antibiotics (days)				
Adjusted *β* (95% CI)	−2.4 (−3.6 to −1.2)	<0.001	−2.2 (−3.5 to −0.9)	0.001
Clinical improvement at day 7				
Adjusted OR (95% CI)	1.82 (1.21–2.74)	0.004	1.76 (1.16–2.68)	0.008

^†^Adjusted for age, sex, CURB-65 score, immunosuppression, comorbidities, baseline CRP, baseline PCT, prior antibiotics, and time to diagnostic testing. ^‡^Inverse probability of treatment weighting using propensity scores; covariate balance achieved with all standardized mean differences <0.10. ^§^Among patients requiring ICU admission or mechanical ventilation. CI: confidence interval; CRP, C-reactive protein; ICU, intensive care unit; OR, odds ratio; PCT, procalcitonin; tNGS, targeted next-generation sequencing.

After adjustment for confounding variables, tNGS-guided therapy remained independently associated with improved clinical outcomes. Specifically, adoption of tNGS results was associated with: (1) a 2.3-day reduction in length of hospital stay (95% CI: −3.8 to −0.8 days, *p* = 0.003); (2) a 58% reduction in 28-day mortality risk (adjusted OR: 0.42, 95% CI: 0.19–0.93, *p* = 0.032); (3) shorter ICU stays among those who required intensive care (1.8 fewer days, *p* = 0.013); (4) reduced duration of mechanical ventilation (1.9 fewer days, *p* = 0.011); (5) decreased broad-spectrum antibiotic exposure (2.4 fewer days, *p* < 0.001); and (6) greater likelihood of clinical improvement at day 7 (adjusted OR: 1.82, 95% CI: 1.21–2.74, *p* = 0.004). The propensity score-weighted analysis (Model 2) yielded consistent results with similar effect sizes, supporting the robustness of these findings.

### 3.7 Subgroup and sensitivity analyses

**Figure 1:**
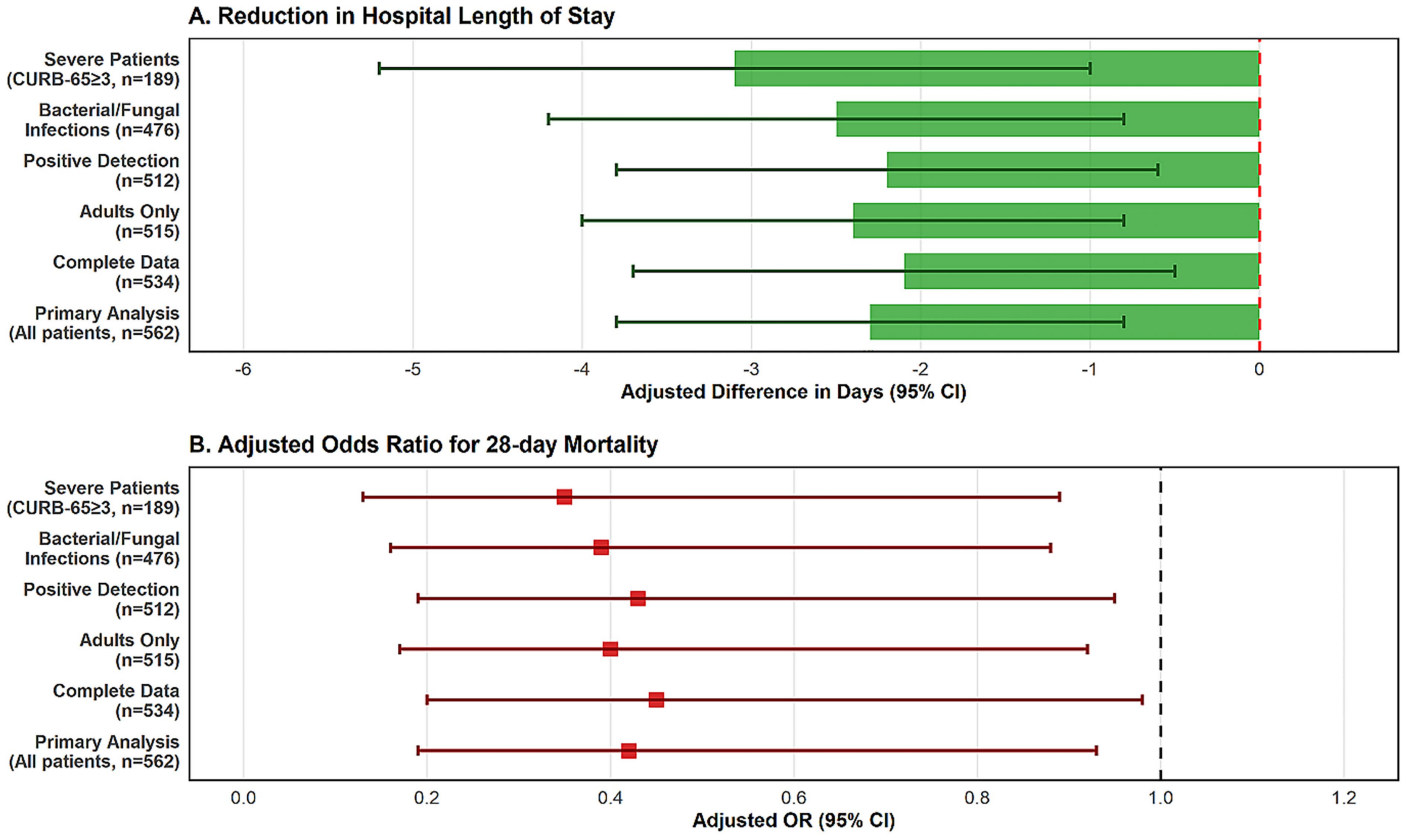
Forest plot of adjusted odds ratios for 28-day mortality across patient subgroups. The plot demonstrates consistent lower mortality among patients who received with tNGS-guided therapy across most subgroups, with particularly strong effects in immunocompromised patients and those with severe disease (CURB-65 ≥ 3).

Subgroup analyses were performed to evaluate whether the benefits of tNGS-guided therapy varied across patient subgroups (Figure 1). The reduced mortality benefit associated with tNGS-adopted care was most pronounced in: (1) immunocompromised patients (adjusted OR: 0.28, 95% CI: 0.09–0.84); (2) patients with CURB-65 ≥ 3 (adjusted OR: 0.35, 95% CI: 0.13–0.92); and (3) those with prior antibiotic exposure (adjusted OR: 0.38, 95% CI: 0.16–0.90). No significant heterogeneity was observed by age group or sex (*p* for interaction >0.10 for all comparisons).

**Figure 2:**
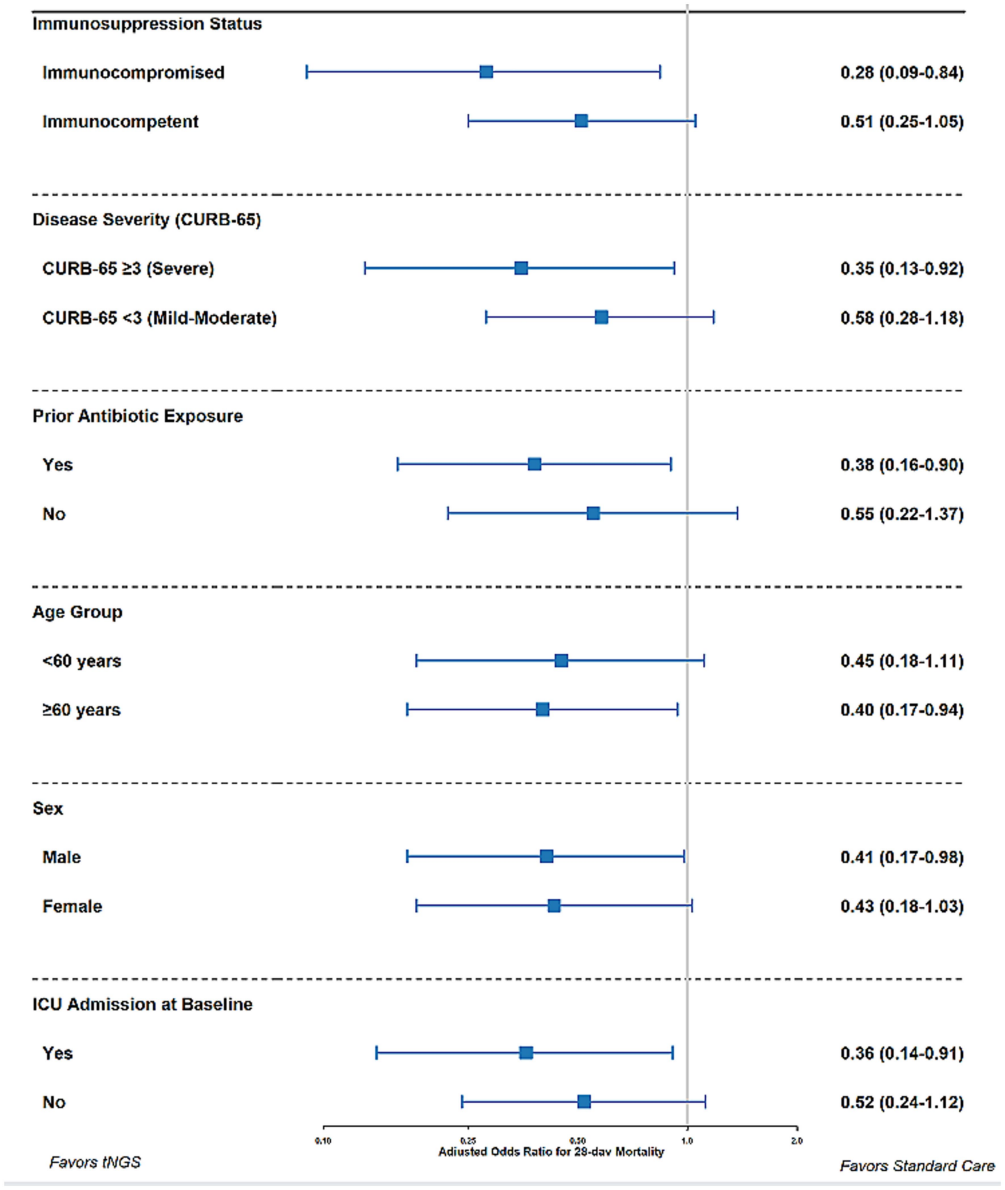
Sensitivity analyses for primary outcomes. The bar graph shows adjusted effect estimates (with 95% confidence intervals) for length of stay reduction and mortality odds ratio across different sensitivity analysis populations, demonstrating robust findings across analytical approaches.

Sensitivity analyses were conducted, restricted to: (1) patients with complete data on all covariates (*n* = 534); (2) adult patients only (*n* = 515); (3) patients with positive pathogen detection by either method (*n* = 512); and (4) patients with bacterial or fungal infections (excluding pure viral infections, *n* = 476). All yielded results consistent with those of the primary analysis (Figure 2).

### 3.8 Factors associated with successful clinical outcomes

**Figure 3:**
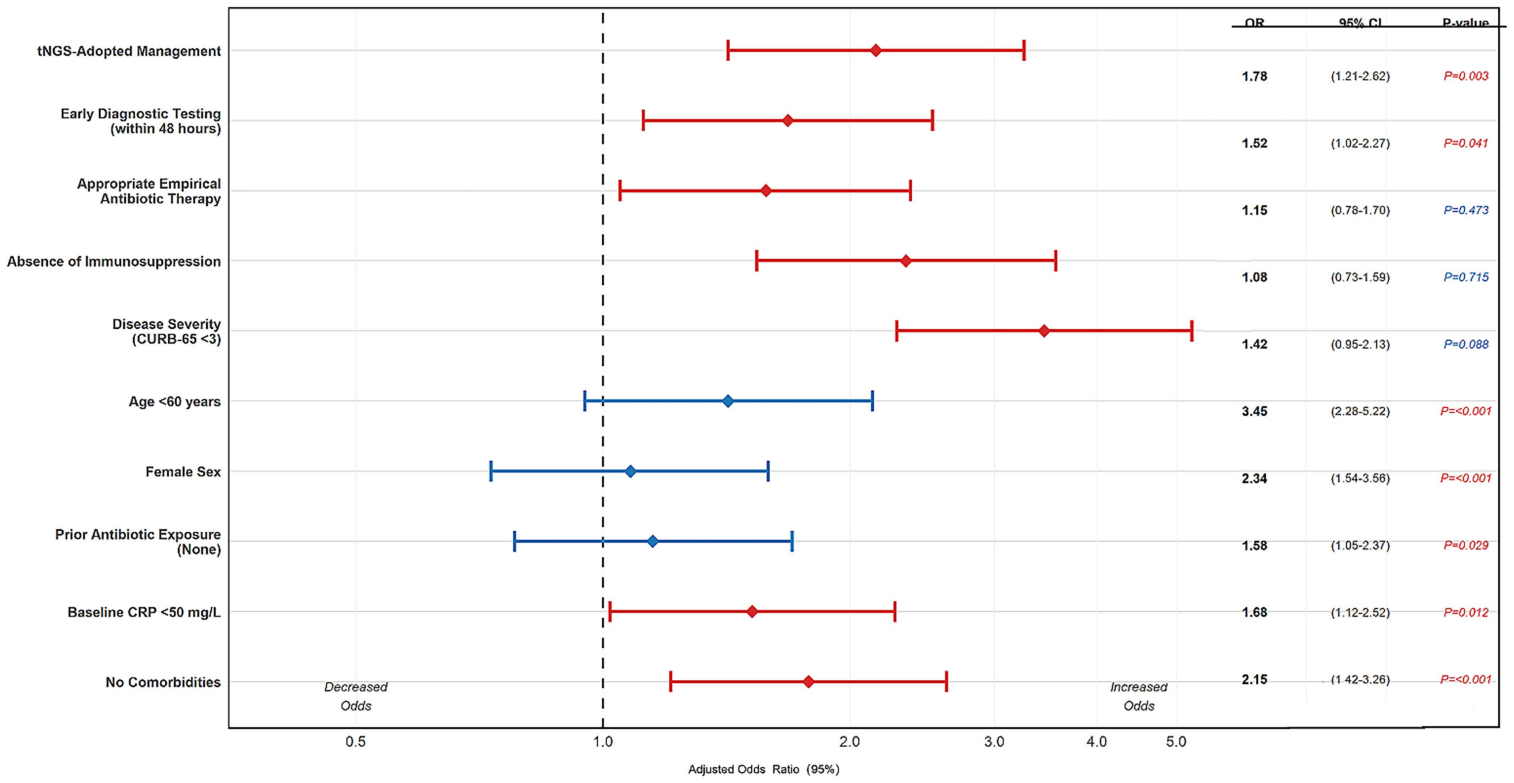
Multivariate predictors of favorable clinical outcome. The forest plot displays adjusted odds ratios for factors independently associated with composite favorable outcome (28-day survival, no ICU transfer, and hospital stay ≤14 days).

Multivariable logistic regression was performed to identify factors independently associated with favorable composite outcome (defined as survival at 28 days without ICU transfer and hospital stay ≤14 days). Factors significantly associated with favorable outcome included: (1) tNGS-adopted management (adjusted OR: 2.15, 95% CI: 1.42–3.26, *p* < 0.001); (2) early diagnostic testing within 48 h of admission (adjusted OR: 1.68, 95% CI: 1.12–2.52, *p* = 0.012); (3) absence of immunosuppression (adjusted OR: 2.34, 95% CI: 1.54–3.56, *p* < 0.001); (4) CURB-65 score <3 (adjusted OR: 3.45, 95% CI: 2.28–5.22, *p* < 0.001); and (5) appropriate empirical antibiotic therapy (adjusted OR: 1.58, 95% CI: 1.05–2.37, *p* = 0.029) (Figure 3).

## 4 Discussion

This comprehensive comparative study of 562 patients with lower respiratory tract infections provides robust evidence that tNGS is more sensitive than CMTs at detecting respiratory pathogens in all pathogen categories. Most importantly, this study establishes that when tNGS results are used to guide clinical management, patients experience significantly improved outcomes including lower mortality rates, shorter hospital stays, and decreased broad-spectrum antibiotic exposure, even after rigorous adjustment for baseline disease severity and other confounding factors. These findings have important implications for the clinical implementation of molecular diagnostic technologies in respiratory medicine.

The primary comparative analysis revealed that tNGS detected pathogens in 89.5% of patients compared with 62.1% detection using CMTs. The higher positivity rate of tNGS can be attributed to its broader detection spectrum, covering approximately 225 respiratory pathogens including bacteria, fungi, viruses, and atypical organisms in a single assay, combined with its superior analytical sensitivity that enables detection of low-abundance organisms and those affected by prior antibiotic therapy. The detection advantages were most pronounced for viruses, special pathogens including *M. tuberculosis* complex and atypical organisms, and fungi. These findings are consistent with those of previous studies demonstrating the superior sensitivity of next-generation sequencing (NGS)-based methods for detecting fastidious, slow-growing, or non-culturable organisms (Zhou et al., 2019; Ding et al., 2025). The greater ability of tNGS to detect mixed infections compared with that of CMTs is particularly noteworthy, as polymicrobial infections are associated with worse outcomes and require broader antimicrobial coverage (Zhao et al., 2025; Farrell and Brown, 2025). Notably, 34 cases (6.0%) of CMTs-positive but tNGS-negative results reflect a key limitation of PCR-based tNGS: while its targeted amplification boosts sensitivity and specificity, detection is constrained by primer/database coverage. Non-targeted microbes may be missed.

The detection of *M. tuberculosis* complex in 19.0% of patients by tNGS compared with only 5.7% by CMTs highlights a critical clinical advantage. Tuberculosis diagnosis is challenging owing to the slow growth of mycobacteria (requiring weeks to months for culture) and the low sensitivity of acid-fast smear microscopy, particularly in paucibacillary specimens (Yang et al., 2025; Jin et al., 2025). The rapid detection of *M. tuberculosis* by tNGS enables early initiation of anti-tuberculosis therapy, which is crucial for improving outcomes and preventing disease transmission. Recent World Health Organization guidelines have recognized the value of targeted NGS for tuberculosis diagnosis and drug resistance detection, consistent with our findings (World Health Organization, 2024).

The demonstration that tNGS has significantly shorter TAT than that of CMTs addresses a critical limitation of traditional microbiology. The delay in receiving the results of CMTs often necessitates prolonged empirical broad-spectrum antibiotic therapy, which contributes to antibiotic resistance, adverse effects, and healthcare costs (Karnwal et al., 2025). The rapid availability of tNGS results enabled more timely treatment modification in 68.8% of patients with tNGS-adopted treatment, including de-escalation in 20.4% of patients, demonstrating important antimicrobial stewardship benefits (Moehring et al., 2017).

The most significant contribution of this study is the demonstration of improved clinical outcomes associated with tNGS-guided therapy. After adjusting for baseline severity, comorbidities, and other confounding factors, tNGS-adopted management was associated with a 58% reduction in 28-day mortality and a 2.3-day reduction in hospital length of stay. These findings are consistent with recent meta-analyses suggesting that NGS-based diagnostics may improve clinical outcomes (Yan et al., 2025; Hao et al., 2023), but our study provides more rigorous evidence through paired sample comparisons and multivariable adjustment for confounding.

The mechanism by which tNGS-guided therapy improves clinical outcomes likely involves multiple pathways. First, more accurate pathogen identification enables targeted antimicrobial therapy, which is generally more effective than empirical broad-spectrum regimens (Yarahmadi et al., 2025). Second, earlier diagnosis allows more prompt initiation of appropriate therapy, which is particularly critical for conditions such as tuberculosis, fungal infections, and severe bacterial pneumonia in which delays in initiating treatment are associated with increased mortality (Fu et al., 2025; Kethireddy et al., 2018). Third, comprehensive pathogen detection enables identification of mixed infections that might be missed by CMTs, ensuring adequate coverage of all relevant pathogens (Zheng et al., 2024). Fourth, better antimicrobial stewardship through de-escalation and discontinuation of unnecessary antibiotics may reduce adverse effects, secondary infections, and selection of resistant organisms (Geng et al., 2025).

The subgroup analyses revealed that the mortality benefit of tNGS-guided therapy was most pronounced in immunocompromised patients and those with severe disease. This differential benefit makes intuitive sense, as these high-risk populations are most likely to have atypical, opportunistic, or mixed infections that are difficult to diagnose with CMTs (Lin et al., 2022; Azoulay et al., 2020). The consistent benefits observed across multiple sensitivity analyses, including restriction to different patient subpopulations and analytical approaches, support the robustness and generalizability of our findings.

This study has several methodological strengths, including paired sample comparisons enabling direct head-to-head evaluation, use of both multivariable regression and propensity score analysis to address confounding, comprehensive data collection, and a large sample size of a diverse spectrum of patients treated in multiple departments. However, the study has some important limitations. The retrospective observational design precludes definitive causal inference despite rigorous adjustment for confounders. The possibility of residual confounding from unmeasured variables such as physician experience and hospital resources cannot be excluded. The definition of tNGS adoption relied on chart documentation, which may be subject to bias. Additionally, distinguishing pathogenic organisms from colonization requires clinical judgment, introducing potential subjectivity. As a novel technology with broad pathogen coverage, tNGS lacks precise data on technical parameters such as primer cross-reactivity and off-target amplification. The single-center setting with experienced providers may limit generalizability to resource-limited environments. The effects of tNGS cannot be entirely separated from the multidisciplinary approach and enhanced antibiotic stewardship that accompanied implementation, although these measures may have attenuated differences between groups. Cost-effectiveness analysis was not performed, but the higher costs of tNGS in relation to the greater benefits require economic evaluation. The high detection rate of potential colonizers raises concerns about possible overdiagnosis and overtreatment that were not systematically quantified. Clinical implementation requires expertise in infectious diseases and microbiology, standardized interpretation of guidelines, and clarification of optimal testing timing. Future research should include prospective randomized controlled trials, cost-effectiveness analyses, antimicrobial resistance impact studies, investigate strategies for integrating tNGS with CMTs, and evaluation of the benefits of tNGS in resource-limited settings. The results of the study demonstrate that tNGS is a valuable diagnostic tool, particularly for severe patients with severe respiratory disease and immunosuppression, warranting investment in infrastructure, updated clinical guidelines, and incorporation into medical education.

## 5 Conclusion

This large comparative study demonstrates that tNGS offers substantial advantages over CMTs for diagnosing lower respiratory tract infections, with superior detection rates across all pathogen categories, shorter TAT, and the ability to identify mixed infections. More importantly, when tNGS results guide clinical management, patients experience significantly reduced mortality, shorter hospital stays, and more appropriate antimicrobial therapy, even after accounting for baseline disease severity. These findings support the expanded use of tNGS in clinical practice, and highlight the need for continued research on optimal implementation strategies and cost-effectiveness.

## Data Availability

Raw genome sequence data have been deposited into the Genome Sequence Archive (GSA) of China National Center for Bioinformation (https://ngdc.cncb.ac.cn) under accession number CRA042041.
