# Supplementary material for: Comparative study of targeted next-generation sequencing and traditional pathogen detection methods in lower respiratory tract infections: impact on patient outcomes
Source: Front Microbiol. 2026 May 8;17:1802040. doi: 10.3389/fmicb.2026.1802040 (PMC13194471; doi:10.3389/fmicb.2026.1802040)
Supplement: Supplementary file 1 [file Table_1.docx]

**Supplementary Material 1 The panel of tNGS detection for common respiratory pathogens**

| Category | Species Name | | |
| --- | --- | --- | --- |
| Gram-positive bacteria（51） | *Streptococcus pneumoniae*  *Streptococcus pyogenes*  *Streptococcus agalactiae*  *Nocardia anthracis*  *Nocardia abscess*  *Nocardia guinea pig otitis*  *Neonocardia*  *Micromonas aeruginosa*  *Streptococcus intermedius*  *ontuberculosis mycobacteria*  *Intracellular Mycobacterium*  *Mycobacterium abscess*  *Mycobacterium boehlii*  *Marsella mycobacterium*  *Mycobacterium abscessus subsp. abscessus*  *Mycobacterium Gordon*  *Mycobacterium scrofulum*  *Mycobacterium szulgai*  *Mycobacterium smegmae* | *Mycobacterium tuberculosis complex*  *Staphylococcus aureus*  *Staphylococcus lugdunensis*  *St. George's Nocardia*  *Star-shaped Nocardia*  *Terpene Nocardia*  *Streptococcus agalactiae*  *Streptococcus anginosus group*  *Arcanobacterium pyogenes*  *Mycobacterium avium complex*  *Turtle abscess mycobacterium complex*  *Mycobacterium asiaticum*  *Mycobacterium Kansas*  *Mycobacterium schneiderii*  *Mycobacterium bufonis* | *Corynebacterium diphtheriae*  *Nocardia genus*  *Nocardia transvalensis*  *Brazilian Nocardia*  *Concave Nocardia*  *African Nocardia*  *Whipple disability body*  *Streptococcus pharyngitis*  *Streptococcus equi*  *Mycobacterium avium*  *Mycobacterium turtle*  *Hidden Mycobacterium*  *Mycobacterium malmoense*  *Mycobacterium apei*  *Occasional Mycobacterium*  *Corynebacterium striatum*  *Listeria monocytogenes bacteria Lister* |
| Gram-negative bacteria（41） | *klebsiella pneumoniae*  *Klebsiella oxytoca*  *Klebsiella pneumoniae*  *Klebsiella pneumoniae*  *Pseudomonas aeruginosa*  *Burkholderia cepacia*  *Burkholderia polyphagia*  *Escherichia coli*  *Katamorella*  *Acinetobacter Ursin*  *Hombutella*  *Legionella bozemanii*  *Neisseria meningitidis* | *Serratia marcescens*  *Proteus mirabilis*  *Stenotrophomonas maltophilia*  *Haemophilus influenzae*  *Elizabethkingia spp.*  *Anopheles elizabethrum*  *Burkholderia cepacia*  *Burkholderia anthracis*  *Meningeal sepsis Elizabethan bacteria*  *Acinetobacter baumannii*  *Acinetobacter calcoaceticus-baumannii complex*  *Bordetella pertussis*  *Legionella genus*  *Legionella longbeachae*  *Duo Sha Pasteurella* | *Necrotizing Clostridium*  *Fusobacterium nucleatum*  *Fragile Bacteroides*  *Brucella genus*  *Onion Burkholderia complex*  *Polluted Burkholderia*  *Burkholderia pseudomallei*  *Enterobacterium cloacae complex*  *Acinetobacter junii*  *Bordetella pertussis*  *Legionella pneumophila*  *Legionella micdadei*  *Citrobacter freundii* |
| DNA virus（42） | *Herpes simplex virus type 1*  *EB virus*  *Human Herpesvirus 6A*  *Human parvovirus B19*  *Human Bocavirus*  *Human Boca virus type 3*  *Human adenovirus group A*  *Human adenovirus type 31*  *Human adenovirus type 7*  *Human adenovirus type 55*  *Human adenovirus type 2*  *Human adenovirus type 8*  *Human adenovirus type 28*  *Human adenovirus group E*  *WU polyomavirus* | *Herpes simplex virus type 2*  *Cytomegalovirus*  *Human Herpesvirus 6B*  *Human Boca virus type 1*  *Human Boca virus type 4*  *Human adenovirus type 12*  *Human adenovirus group B*  *Human adenovirus type 21*  *Human adenovirus group C*  *Human adenovirus type 5*  *Human adenovirus type 24*  *Human adenovirus type 30*  *Human adenovirus type 4*  *BK Polyomavirus* | *Varicella zoster virus*  *Human Herpesvirus 6*  *Human Herpesvirus 7*  *Human Boca virus type 2*  *Human adenovirus*  *Human adenovirus type 18*  *Human adenovirus type 3*  *Human adenovirus type 34*  *Human adenovirus type 1*  *Human adenovirus group D*  *Human adenovirus type 27*  *Human adenovirus type 38*  *JC Polyomavirus* |
| RNA virus（45） | *Human parainfluenza viruses*  *Human respiratory virus type 1*  *Human mumps virus type 4*  *Enterovirus A71*  *Coxsackie virus A6 type*  *Enterovirus Group B*  *Ebola virus E30 type*  *Enterovirus D68*  *Human Coronavirus OC43*  *Rhinovirus type A*  *measles virus*  *Mumps virus*  *H1N1 influenza A virus*  *Influenza A virus H7N9*  *Yamagata strain of influenza B virus*  *Human respiratory syncytial virus type B* | *Human mumps virus type 2*  *Enterovirus*  *Coxsackie virus A2 type*  *Coxsackie virus A10*  *Coxsackie virus B3 type*  *Enterovirus Group C*  *Human Coronavirus HKU1*  *Human Coronavirus 229E*  *Rhinovirus type B*  *Rubella virus*  *Influenza A virus*  *Influenza A virus H3N2*  *Influenza B virus*  *Influenza C virus* *Novel coronavirus Disease-2019* | *Human respiratory virus type 3*  *Enterovirus Group A*  *Coxsackie virus A5 type*  *Coxsackievirus A16*  *Escherichia coli E18 type*  *Enterovirus Group D*  *Human Coronavirus NL63*  *Rhinovirus*  *Rhinovirus type C*  *Human leaning lung virus*  *H1N1 influenza A virus (2009)*  *Influenza A virus H5N1*  *The Victoria strain of influenza B virus*  *Human respiratory syncytial virus type A* |
| Fungus （44） | *Candida albicans*  *Near smooth Candida*  *Pichia guilliermondii*  *Cryptococcus gattii*  *Irregular Mucor*  *Aspergillus fumigatus*  *Aspergillus terrestris complex group*  *Pseudomonas aeruginosa*  *Branched Aspergillus*  *Rhizopus microsporus*  *Rhizomucor pusillus*  *Syncephalastrum* | *Tropical Candida*  *Candida glabrata*  *Aspergillus asaci*  *Marneffei basket shaped bacteria*  *Mycoplasma membranaceus*  *Aspergillus flavus complex group*  *Sedosporium genus*  *Aspergillus genus*  *Rhizopus genus*  *Rhizopus oryzae*  *Fusarium genus*  *Aspergillus*  *Cunninghamella*  *Microascus*  *Cryptococcus*  *Cryptococcus laurentii* | *Pseudosmooth Candida*  *Kudriazwei Pichia pastoris*  *Cryptococcus neoformans*  *Mucor racemosus*  *Pneumocystis jiroveci*  *Aspergillus niger complex*  *Aspergillus cuspidatum*  *Umbelliform Aspergillus*  *Rhizopus delemar*  *Rhizopus genus*  *Lomentospora prolificans*  *Candida auris*  *Mucor*  *Candida genus*  *Coccidioides* |
| Mycoplasma/ Chlamydi/ Rickettsia/Parasite（10） | *Mycoplasma pneumoniae*  *Chlamydia psittaci*  *Ureaplasma parvum* | *Chlamydia pneumoniae*  *Chlamydia trachomatis* | *Coxiella burnetii*  *Ureaplasma urealyticum*  *Paragonimus westermani*  *Leptospira*  *Mycoplasma hominis* |
